# Development and validation of a PLE scale from academic administrative perspective (PLES-AA) in tertiary education: A pilot study in China

**DOI:** 10.1371/journal.pone.0272214

**Published:** 2022-08-05

**Authors:** Xiaoshu Xu, Kimberly Kolletar-Zhu, Jia Liu, YunFeng Zhang, Na Cai

**Affiliations:** 1 School of Foreign Languages, Wenzhou University, Wenzhou, Zhejiang Province, China; 2 Department of Foreign Studies, Zunyi Medical University, Zhuhai Campus, Zhuhai, Guangdong Province, China; 3 Centre for Portugese Studies, Macau Polytechnic University, Macau, Macao; 4 Vocational Education Teachers Institution, Guangdong Polytechnic Normal University, Guangzhou, Guangdong Province, China; Public Library of Science, UNITED KINGDOM

## Abstract

The study aims to construct and validate a rubric to assess the effectiveness of PLEs from an academic administrative perspective (PLER-AA) in tertiary education in China. A qualitative-quantitative sequential mixed-method design was used for the scale validation. A total of 206 teachers and administrative staff participated in the Confirmative Factor Analysis (CFA), which supported the 4-dimensional scale, with policy (n = 4), program design (n = 4), curriculum/instruction (n = 4), and capacity (n = 4). Meanwhile, another 189 teachers and administrative staff participated in the current sequence of PLE applications in higher education surveys, revealing a developing phase in China. Consequently, the rubric can be used as a benchmark that provides insight to educators and administrators in developing PLEs in tertiary education in China and worldwide.

## 1. Introduction

Higher Education is transitioning from a transmission model of education toward one based on active learning, personalisation, hybrid course designs, and innovative ways to track degree progress. Personal Learning Environments (PLE) represent a ground-breaking new development in educational practices that incorporates Information and Communications Technology (ICT) and an opportunity to support the building of universities without boundaries that can meet the needs of the knowledge society [1].

**Fig 1:**
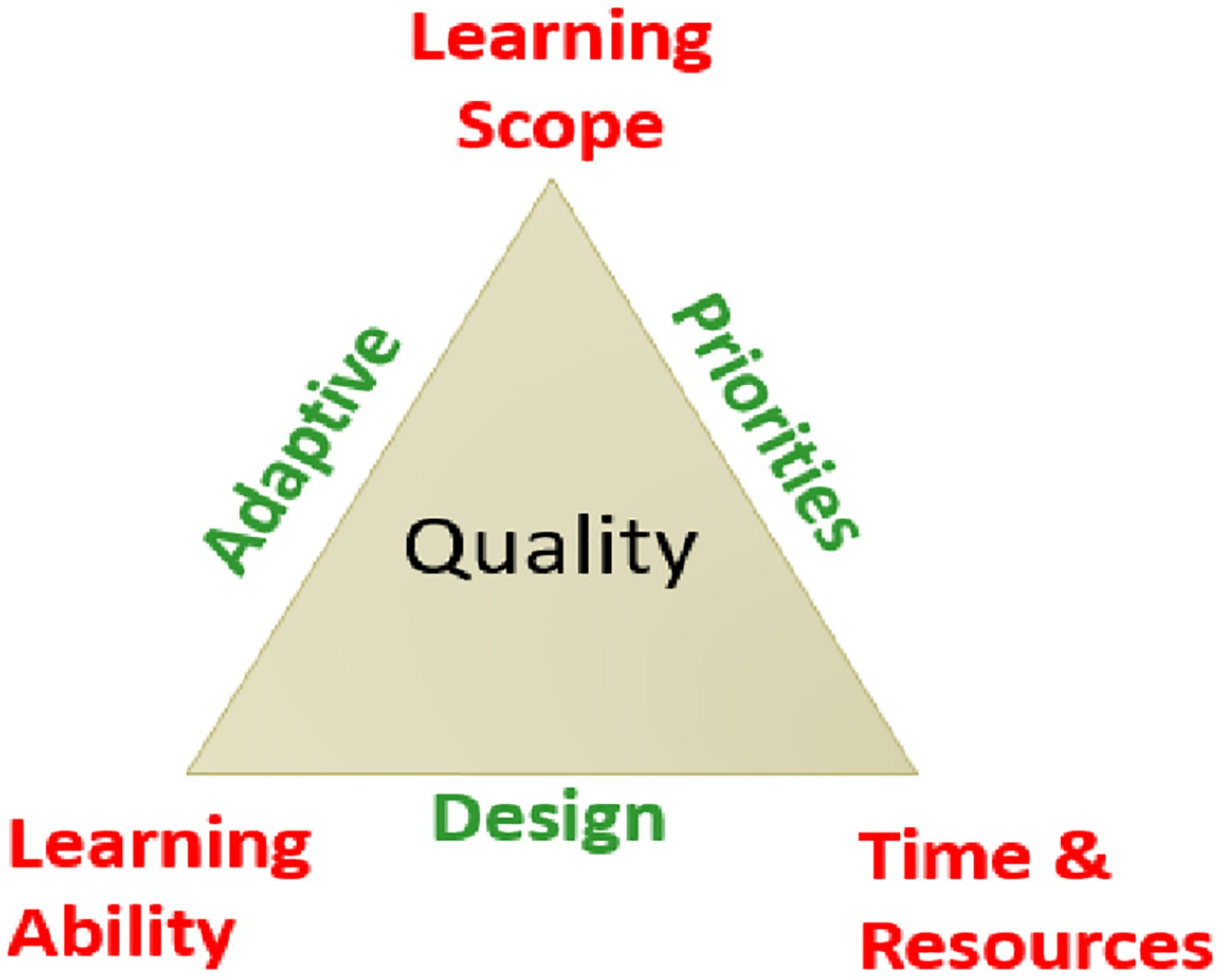
Pedagogical perspective of PLE in formal education.

However, PLEs are undoubtedly one of the most disruptive approaches in the field of educational technology in recent years [2–4]. For one thing, higher education has its own set of goals, requirements, and regulations; [5, 6] for another, PLE has its own set of principles, characteristics, and guidelines, some of which are in direct conflict with one another. (See Fig 1 below).

As the above figure shows, firstly, formal education courses have their learning scope; secondly, each course has specific resource-bound real-time processes including learning schedule, course goals, teaching plans, teaching material, etc.; thirdly, learners with different learning backgrounds differ in cognitive processes and characteristics which need additional teachers’ supervision or guidance. Besides, each side of the triangle represents the relationship between two angles. Learning scope and ability, for example, require learners to use appropriate learning strategies to select appropriate learning content; learning ability and time demands assist learners in developing specific, measurable, attainable, relevant, and time-bound plans; and learning scope and time require learners to prioritize learning resources.

As a result, PLEs may have to give up some of their personalization features in order to bridge the gap between them and higher education. Simultaneously, higher education may see a paradigm shift from teacher-directed to learner-centred, from one-size-fits-all to a range of learning experiences, and so on (see Table 1 below).

**Table 1:** Six key factors of PLE vs higher education.

PLE	Higher education
Learner-Centered based on learning needs	Teacher-Directed based on the school curriculum
Variety of Learning Interactions	Mainly One-Size-Fits-All
Technology to Transform	Technology to Enhance
Data Impacts and Informs Learning	Data Evaluates Teaching & Learning
Competency-Driven	Pacing-Chart-Driven
Collaboration with Peers and Community	Mainly Independence

Source: self-made (summarized from literature review)

Because PLEs are made up of five main elements: learning profiles, learning networks, learning tools, learning resources, and learning services, [7] integrating PLEs with higher education necessitates adjusting these five elements from four different perspectives: academic administration, teacher & teaching, learner & learning, and technology. (see Table 2 below).

**Table 2:** Converge PLE with higher education.

	Academic administration	Teacher & teaching	Learner & learning	Technology
**Learning profile**	Setting	Verify	Manage	Auto Capture
**Learning network**	Monitor	Assist	Participate	Provide Implement
**Learning tools**	Recommend	Modify	Utilize	Integrate
**Learning resources**	Accredit	Select	Explore	Adopt
**Learning service**	Comply to	Collaborate	Follow	Fulfill

Source: self-made (summarized after consulting the ICT and academic administrative experts)

Academic administration is the most important of the four perspectives. Higher education administrations must address the contradiction that exists between the emphasis on standardized testing and assessment regimes in higher education and the foundation of PLE, which is built on learner autonomy and potential realization. Without fundamental pedagogical reforms, the PLE applications could not be successful. Academic administrative practices must be reshaped and reorganized when PLEs are implemented. Set appropriate learning profiles, [8] for example, monitor learners’ learning networks, [9] recommend appropriate learning tools, [10, 11] accredit learning resources, and provide learning services [12, 13].

The literature review, on the other hand, revealed that the study of academic administration in online learning is still in its infancy. It is critical to develop an assessment instrument to judge the efficacy of a PLE platform and how to best use it in a tertiary setting. Although the other three perspectives are important, they are beyond the scope of this study’s research. The goal of this study was to create and validate a scale for evaluating PLEs from an academic administrative standpoint in tertiary education in the Chinese context.

## 2. Literature review

### 2.1 PLEs and academic administration

The concept of PLEs tends to drift into techno centrism, usually including the reference to social media and the possibilities for the creation and sharing of knowledge which ultimately relates to the student’s control of learning [14]. Attwell, on the other hand, declared that PLEs were essentially a social pedagogical approach to using technology for learning [15]. In the current study, PLEs are not only technical platforms, but also a new digital learning literacy, conceptual space, pedagogical process, and social networks that enable and support learners in achieving their lifelong learning objectives. PLEs are viewed as dynamic, interconnected environments with a constantly changing community of learners, instructors, tools, and content.

The implementation of PLEs in higher institutions is not only about technology reform, but a philosophy or a way of working as asserted, by Sebba et al. [16] The essence of PLEs is a vision to empower learners to take more ownership of their learning, as well as to foster learner autonomy and lifelong learning [17]. Academic administration, which was often able to bring a broader perspective to bear on issues of the day, plays a decisive role in this paradigm shift [18].

Previous studies, on the other hand, have done little research on academic administration, and the majority are decades old. For instance, Sang et al. [19] developed a generalized goal-programming model for analyzing resource-allocation decisions for academic administrators. To create effective conditions for personalized learning, Underwood et al. [6] identified the need for a synergy of investment and opportunity within four nested educational spaces (school/institution, teaching space, personal learning space, and living space). For example, designing adaptive PLEs necessitates massive human knowledge input as well as working through the coding and algorithm decisions that will make PLEs a reality. These investments and opportunities would be difficult to find if higher education institutions remained stable and resistant to change. Donovan and McFarlane identified activities in which distance education managers can demonstrate exemplary leadership functions using Mintzberg’s theory of information, interpersonal, and decision management roles and activities. to identify activities in which distance education managers can display exemplary leadership functions. PLEs were identified as important academic administrative and instructional strategies for empowering students to become competent digital lifelong learners [20].

### 2.2 A review of academic administrative scales for online education

According to the literature reviews, there are no published PLE Scales from the Academic Administrative perspective (PLES-AA). As a result, this research focuses on academic administrative scales for online education. The concept of institutionally-powered PLEs (iPLEs) did, however, shed some light on this research.

iPLEs are pre-configured digital environments created by institutions that enable pre-formed by the institutions that allow students to create and organize their networks of learning resources, applications, and tools based on their interests and objectives, as well as communicate with others participating in specific learning activities [21–24].

As previously stated, an operational application of PLEs in higher education necessitates academic administration, which includes policy development, support to organizations and internal quality assurance systems, professional development activities, technological support systems, and so on. In the last fifteen years, a review of Social Science Citation Indexed papers on online education rubrics and frameworks from a pedagogical perspective yielded 27 studies. For example, the “Personal Learning Environments Questionnaire” by Martinez et al. [25] explained the management and planning of PLE. The “Quality Assurance Agency in Scotland, Enhancement Themes initiative (2014–17)” Adekola et al., [26] indicated four aspects of pedagogy, namely stakeholders’ expectations, management and organisation. institutional culture and ethics and legal. The “Administration Considerations Impacting the Quality of Online Teaching” by Hammond et al. [27] demonstrated four categories of pedagogy, including faculty support, better communication, student support, and faculty communication. However, the majority of the research focused on pedagogical methods and teaching practices. For example, Adekola et al. developed an institutional framework to guide transitions to enhanced blended learning in higher education [26]. Gordon and the Higher Education Academy proposed flexible pedagogies in technology-enhanced learning [28].

Meanwhile, a review of benchmarks, rubrics, and frameworks for quality distance education or online education was conducted, including the Quality on the Line developed by the Institute of Higher Education Policy in Washington, DC, [29] the ACODE [30] developed by the Australasian Council on Open Distance and e-Learning, and the Quality Assurance Framework developed by the Asian Association of Open Universities (AAOU) [30–32] (see Table 3 below). However, each of those benchmarks had advantages in a specific context.

**Table 3:** Benchmarks/rubrics/framework for quality online education.

Institution & year	Name of scale	Categories	Reference location
WASC 2011	Rubric for Assessing Student Learning into Program Reviews	Planning & Budgeting Annual Feedback	https://teaching.berkeley.edu/sites/default/files/wasc_genedassesment_1.pdf
QM Quality Matters	Rubric Standards	Managing professional development Course Standards Evaluation Criteria Course technology Accessibility & Usability	https://www.qualitymatters.org/…/files/PDFs/StandardsfromtheQMHigherEducationRubric.pdf
Tennessee Instructional Leadership Standards (TILS)	Administrator Evaluation Rubric	Data Analysis & Use Student Interventions Progress Monitoring Leveraging Educator Strengths Induction, Support, Retention, & Growth	https://team-tn.org/wp-content/uploads/2013/08/TEAM-Admin-Evaluation-Rubric-20161.pdf
Quality on the Line 2000	Benchmarks For Success In Internet-Based Distance Education	Institutional Support Benchmarks Course Development Benchmarks Teaching/Learning Benchmarks Course Structure Benchmarks Student Support Benchmarks Faculty Support Benchmarks Evaluation and Assessment Benchmarks	https://www.ihep.org/publication/quality-on-the-line-benchmarks-for-success-in-internet-based-distance-education/
CHEA Quality Assurance Toolkit for Distance Higher Education 2009	Framework for Quality Assurance in Open and Distance Learning	Development of Performance Indicators Vision, mission and planning Management, leadership and organizational culture Human resource and development Programme design and development Course design and development Learner support Learner assessment Infrastructure and learning resources Research consultancy and extension services Institutional planning and Management	https://open.saide.ngo/repository/opensaide/
The ACODE (Australia) Australasian Council on Open Distance and e-Learning 2014	The ACODE benchmarks have been developed to assist institutions in their practice of delivering a quality technology enhanced learning experience.	Institution-wide policy and governance for technology enhanced learning; Planning for institution-wide quality improvement of technology enhanced learning; Information technology systems, services and support for technology enhanced learning; The application of technology enhanced learning services; Staff professional development for the effective use of technology enhanced learning; Staff support for the use of technology enhanced learning; Student training for the effective use of technology enhanced learning; Student support for the use of technology enhanced learning.	https://www.acode.edu.au/pluginfile.php/550/mod_resource/content/8/TEL_Benchmarks.pdf
Quality Assurance Framework made by the Asian Association of Open Universities (2020)	Quality Assurance Framework. Assuring quality in policy and planning.	Policy & Planning Internal Management Learners & Learners’ Profiles Infrastructure, Media, and Learning Resources Learner Assessment & Evaluation Research & Community Services Human Resources Learner Support Program Design and Curriculum Development Course Design & Development	https://www.aaou.org/quality-assurance-framework/

Source: self-made

## 3. Research design

### 3.1 Methodology

From an academic administrative perspective, the PLE scale was developed using an exploratory sequential mixed-method approach (PLES-AA). Minor revisions were made based on Creswell and Clark’s scale development framework [33]. The initial PLES-AA item pool was developed using the literature review approach, taking into account the aforementioned rubrics and frameworks. This was followed by the collection of qualitative data through structured interviews and an online focus group meeting.

### 3.2 Procedure

The Virtual Learning Program Rubric was primarily referred to in this study’s first phase, for the literature review section [34]. It combines some characteristics, such as integration implementation, a process for assessing implementation integrity, and alignment or integration with other efforts. The rubrics are divided into four domains: policy, program design, curriculum and instruction, and capacity, with a framework for identifying areas for effective teaching and learning in Virtual Learning Programs. Thus, it guides the Virtual Learning Programs with quality and rigour. Each standard has examples to convey the dimension of fully met standards, partially met standards, developing standards, and beginning standards. A scale was developed to identify the criteria for each level.

In terms of the structured interview, the experts were invited to participate as evaluators via a letter of invitation. The category definition and scale form included ratings and indicators with descriptions, the criteria for evaluating the standards, and a comments column for gathering expert suggestions. The first 33 items were emailed to these experts. They were asked to assess how reflective the item constructs were and whether they addressed all or most of the critical academic administrative issues that arise when using PLEs in higher education.

Six administrative staff members with experience in online higher education were invited to the focus group. The members reviewed each item, discussed whether each item delivered what was originally intended, and improved the wordings.

A Confirmative Factor Analysis was administered to validate the perceived PLES-AA. The final version of the scale was evaluated by the two consultants who originally participated in the initial face validity review.

### 3.3 Subjects

One native English-speaking EFL consultant and one Chinese PLE expert initiated the research and organized the face validity of the first version scale. Both were from a leading university in China. They have 10 years of online EFL teaching experience in China and abroad, and they fully understood the purpose of this research.

Second, for the structured interview in the second draft of the instrument, an "expert sampling" design was chosen. Given the importance of the experts’ opinions in the validation of the instrument, the sample was chosen based on criteria that certify their knowledge, experience, and expertise in the field of study [35]. When selecting the experts, we used the criteria referred to by Escobar-Pérez and Cueva-Martínez’s research: [36] a) Have doctoral-level or PhD academic studies; b) Be a university professor or administrative staff; c) Have experience in online higher education or administration work; (d) Understand the context of Chinese higher education; and (e) Have experience with PLE. The interview included five experts in total. The five experts were from universities of various types (leading universities, a university at provincial level, a Sino-foreign institution, and a foreign university). All of the experts are professors or associate professors, three are female, and all have PhDs.

Six members from various universities in China and abroad participated in the focus group. All of the experts had to have at least two years of online teaching experience and five years or more of administrative experience in tertiary education, either in China or abroad, and they had to be well-versed in PLE.

In terms of the PLES-AA application, all participants who joined were required to be university teachers, administrative staff, or ICT experts, as well as have a basic understanding of PLE.

The informed consent was distributed to all participants in the study via the web-based survey software "WenJuanXing" provided by sojump.com. All survey responses are kept anonymous, and their participation in this study was entirely voluntary. Participants were informed that completing the survey would have no direct individual impact, either in terms of gain or loss, because all data collected would be aggregated and only disclosed in a summarized form.

## 4. Findings and discussion for the construction of the scale

### 4.1 Two consultants’ face validity

Each consultant received the scale via email and provided feedback to improve each standard to ensure clarity and adaptability of the content in the Chinese context. Following the face validity, some terminologies, including "policy," "program," and "competency-based," were redefined in the Chinese context. In China, PLE programs are governed by policies implemented by higher education institutions rather than the government, and the concept of "competency-based" education is only realized at the project level. In China, most universities are still test-oriented. Second, some information was removed, such as "certification" in standard 1.8, because Chinese administrators in higher education institutions are not required to be certified. Third, we changed the theme words "virtual learning," "community," and others to better fit the current research scope and goal. The second version of the scale was created following the editing and changes.

### 4.2 Five experts’ structured interview

The items on the scale were evaluated in the following categories for the five experts’ structured interviews: sufficiency, clarity, consistency, and relevance [35]. The following citations are the main recommendations made by the five experts.

According to the Taiwanese expert, the language of the scale is difficult to read and should refer to the Chinese context. Meanwhile, the scale’s development has a relatively clear theoretical foundation and research purpose. The expression of the research’s purpose reflected on this scale is not particularly clear.

The Hong Kong expert made the following remarks: "The scale appears to be universal, but there are many different types of universities on the mainland, including normal colleges, polytechnic colleges, and technical colleges. It is suggested that the scale provide more options for the various institutions."

According to the Macau expert, some terms should be adapted to the actual university context in China. Second, the descriptions of some sub-standards within each dimension are too academic to present clear meanings; and the expression of standards is constantly changing, resulting in inconsistency. It is recommended that specific standards be written as colloquially as possible when developing the scale so that the target group can easily understand the meaning of the standards.

The expert from North Mainland China tried to explain that the questionnaires and scales should be localized. Each item’s expression should be as close to the Chinese context as possible. Some of the terminologies used cause difficulty reading and even ambiguity. In addition, standard PLE features and platform requirements should be included in the scale.

"Since 2012, there have been many studies on PLE in China," said the expert from South Mainland China, "so it is possible to reconstruct the research system of PLE in combination with the existing local research in China and adjust the questionnaire items according to the actual situation in China." It was also proposed that the number of standards is reduced and that the expressions be more concise.

Furthermore, the experts independently refined the items via email. Finally, 12 items were removed from the scale’s second draft, yielding the third version. To address the remaining changes to the items, a focus group discussion was organized.

### 4.3 Focus group discussion

A focus group consisting of six administrative members was formed to revise the third draft utilizing different comparative scales and more extensive reviews of literature. After two one-hour online discussions, the focus group eventually agreed that the four domains/categories should be retained in the third draft PLE scale (policy, program design, curriculum and instruction, and capacity). For each category, a four-level scale was developed, along with descriptors to identify various levels.

The scale used the following criteria:

- Above-average—All characteristics of the standard are developed and implemented with consistency and reliability.
- Sufficient—Characteristics of the standard are developed and implemented but without consistency or reliability.
- Developing—Characteristics of the standard are being developed.
- Start-up—There is little or no development of the standard and little or no implementation of the standard.

Meanwhile, in response to the expert from Macau’s concern that "the expression of standards is constantly changing, lacking consistency," the focus group provided working definitions for the four categories:

- Policy: The criteria in this section intends to ensure there is both a rational and relevant system for the implementation of a PLE platform consistent with overall HE standards and policies.
- Program Design: The criteria in this section reflect the PLE platform’s mission and goals and address the accessibility and understanding of these to relevant stakeholders, including students, parents, and community members.
- Curriculum/Instruction: The criteria in this section reflect the design and rigour of the PLE platform curriculum policies and the reliability of its implementation.
- Capacity: The criteria in this section reflect the ability and capacity of a PLE platform that can support systematic capacities and infrastructure concurrently. The specific operation or implementation method is not included in the scope of this description.

Moreover, some terminologies of the scale were also defined, for instance:

- HE stakeholders—The primary stakeholders include students, teachers, and the management of the educational institute. Other stakeholders are government, professional bodies, employers, parents, non-teaching staff, and auditors.
- The Program Mission Statement—The Program Mission Statement is a concise statement of the general values and principles which guide the curriculum. It sets a tone and a philosophical position that follows a program’s goals and objectives.
- Capacity—In the context of networks, capacity is the complex measurement of the maximum amount of data that may be transferred between network locations over a link or network path.
- Curriculum/Instruction—What we choose to teach is based on the curriculum. How we teach is the instruction.

Based on these working definitions and the agreement made by the focus group, the following revisions of the third draft were made:

1. Reduce the text, delete by combining, and reformulate the wording of items 1.2,
2. 1.3,1.4,1.5,1.6,1.7,1.8 and 1.9
3. Combine items 2.4 and 2.5.
4. Rewrite items 3.3 and 3.4, and add concepts “learning portfolios” and “learning technology interoperability”.
5. Add “The PLE platform supports and recommends personalized learning tools” as item 4.4.
6. The level description for 3.4, 4.1, 4.2 and 4.3 use percentages to describe the ability and capacity of a PLE platform.

This then generated the fourth version scale which contained four domains with 16 items (see S1 Appendix).

### 4.4 Second-round consultant interview

The fourth version of the scale was sent to the previous two consultants for the second-round review and evaluation [36]. The two consultants provided positive feedback on all 16 items, but they proposed an easy-to-understand definition of PLE as follows:

*The personal learning environment platform is a* "*Learner-centered personal management space*, *where learners are self-regulated to control learning progress*, *participate in various social networks*, *and use a series of network protocols to connect systems and resources*.”

## 5. The confirmative factor analysis of the PLES-AA

### 5.1 Instrument

For the Confirmative Factor Analysis, a questionnaire for the importance of PLE implementation was developed based on the generated PLES-AA, which was composed of two parts: the demographic information, including gender, age, and degree, and 16 validated PLE items in the form of a five-level Likert scale (1 = not important, 5 = very important). A pilot test of 40 questionnaires was carried out to ensure that the questions were formulated clearly and understandably and that the responses generated the necessary information to answer the research questions. To ensure the quality of the answer, respondents suggested adding multiple-choice questions about familiarity with a PLE study. The refined Chinese versions of the questionnaire surveys were distributed via Wenjuanxing, e-mail, QQ, and WeChat.

### 5.2 Participants

Snowball sampling and convenient sampling methods were used. The Wenjuanxing platform was used for the survey, which targeted higher education academic administrators as well as experts and teachers in higher education ICT fields. In the end, 206 valid responses were received.

### 5.3 Demographics

The majority of the 206 participants, 62.6%, were female, with the remaining 37.4% being male; 80% were between the ages of 30–49, with 55.8% holding a Master’s degree, while 38% holding a doctorate; half were affiliated with higher education institutions located in the southern part of China, while 27% were affiliated with higher education institutions in the eastern part of China. Higher education teachers made up 86.9% of the total. The remaining were administrative personnel. 69% of them claim to be familiar with PLE studies.

### 5.4 Results

**Fig 2:**
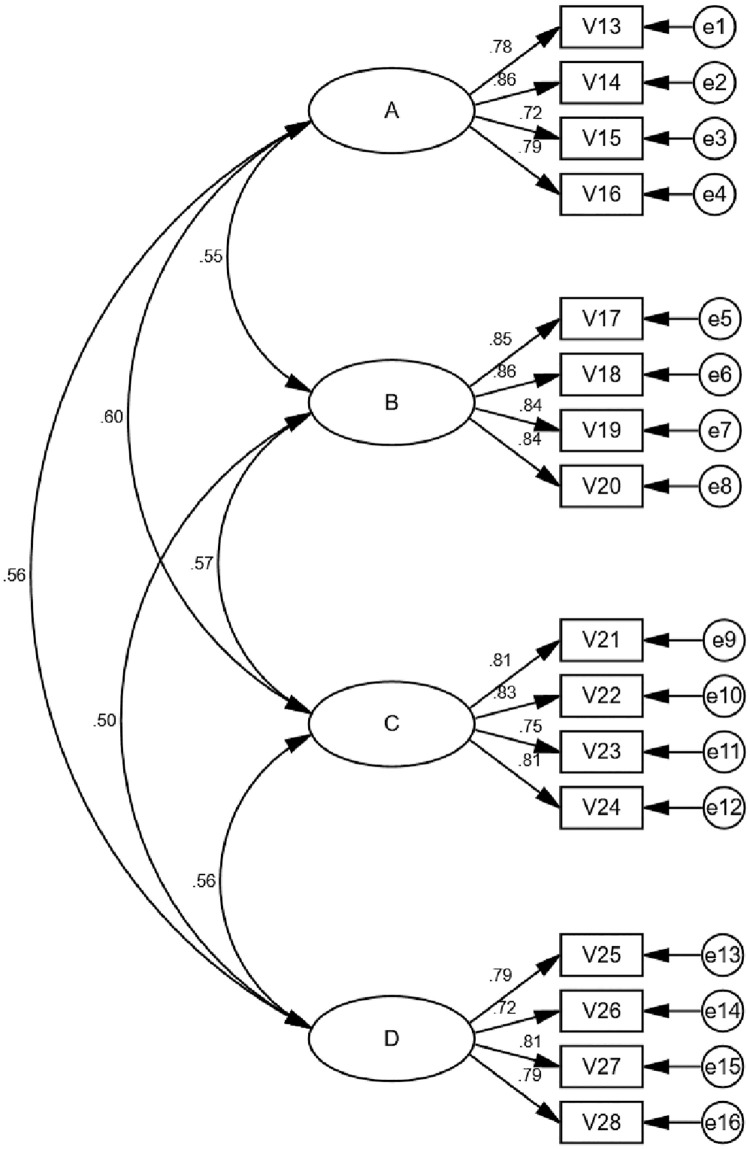
CFA for the PLES-AA scale.

A CFA yields a series of indexes that estimate the extent to which the sample data can fit the a priori assumptions in different ways. In the CFA of this data, 16 items were retained. (See Fig 2). Table 4 shows the fitting indexes corresponding to the model. The values of these indexes indicated that the scale had good structural validity.

**Table 4:** Fitting indexes of the scale.

	CMIN/DF	GFI	AGFI	NFI	IFI	TLI	CFI	RMSEA	SRMR
Value	1.845	0.906	0.869	0.914	0.959	0.949	0.958	0.064	0.052
Threshold	<3	>0.8	>0.8	>0.9	>0.9	>0.9	>0.9	<0.08	<0.08

According to the above table, the X2/df value was 1.845, which was less than 3; RMSEA was 0.064 and SRMR was 0.052, which were less than the standard level of 0.08, indicating a good fit. GFI = 0.906, AGFI = 0.869, NFI = 0.914, IFI = 0.959, CFI = 0.958, TLI = 0.949, all goodness-of-fit indicators met the general criteria, indicating that the validated factor analysis model developed in this study was valid and matched well with the recovered data.

The data collection instrument’s construct validity was assessed using the convergent and discriminant forms of validity. Composite reliability (CR) and Average Variance Extracted (AVE) were calculated in this regard. The value of CR was calculated and the resultant value of all the dimensions was found to be more than 0.80. Whereas the resultant values of AVE ranged from 0.606 to 0.718. The values of CR and AVE were above the threshold value of CR = 0.60 and 0.50 = AVE suggested by Byrne (2016). See Table 5 [37].

**Table 5:** Correlation, validity and reliability of measures.

	CR	AVE	α	A	B	C	D
A	0.866	0.620	0.869	1			
B	0.910	0.718	0.910	.472**	1		
C	0.876	0.639	0.875	.502**	.510**	1	
D	0.860	0.606	0.859	.475**	.439**	.481**	1

**Correlation is significant at the 0.01 level (two-tailed)

The Cronbach’s alpha value was calculated to check the internal consistency and reliability of the 16 scale items. The alpha value was found to be 0.88, above the recommended value of ≥0.70, [38] which indicated good consistency between the various items of the scale. Dimension-wise alpha value was also calculated, as shown in Table 5.

Pearson’s Moment Correlation was applied to determine the relationship between the four dimensions of the PLES-AA. The results revealed that all of the dimensions were positively and significantly correlated with each other at a p-level of 0.01. According to Cohen’s criterion, [39] Policy was strongly correlated with Program Design (r = 0.472**), curriculum and instruction (r = 0.502**), and capacity (r = 0.475**) (see Table 5).

To sum up, the results indicated that the scale had very good reliability and validity, so the PLES-AA could be considered a feasible tool as a quality assurance for academic administrative readiness when implementing PLEs. The final version of the scale is contained in S1 Appendix.

## 6. The application of the PLES-AA

The PLES-AA was applied in the form of a current situation questionnaire survey in China. The questionnaire was similar to the CFA, the only difference was the four-level Likert scale: (4) above average, (3) sufficient, (2) developing, (1) start-up.

### 6.1 Participants

Snowball sampling and criterion sampling methods were used. The WenJuanXing platform was used for the survey, which targeted higher education academic administrators as well as experts and teachers in higher education ICT fields. In the end, 197 valid responses were received. However, eight cases were deleted because the participants were not familiar with a PLE study.

### 6.2 Demographics

The majority of the 189 participants, 66%, were female, with the remaining 34% being male; 78% were between the ages of 30–49, with 61% holding a Master’s degree, while 34% holding a doctorate; half were affiliated with higher education institutions located in the southern part of China, while 33% were affiliated with higher education institutions in the eastern part of China. Higher education teachers made up 88.5% of the total. The remaining were administrative personnel. 66% of them claim to be familiar with PLE studies.

### 6.3 Results

The collected data were analyzed using IBM SPSS25. The median time to complete the questionnaire was around two minutes. To be consistent with the technique utilized by [40] Cronbach’s alpha was used as a measure of the internal consistency of the survey. This refers to the degree to which the items measure the same underlying construct, and a reliable scale should have a Cronbach’s alpha coefficient above 0.70 [41]. The reliability analysis of the instrument yielded a Cronbach’s alpha coefficient = 0.966 and the Kaiser–Meyer–Olkin measure of sampling adequacy (.948). The results suggested the scale had an internal consistency and was reliable. Meanwhile, the approximate chi-square value of Bartlett’s test of sphericity was considerable (3137.883) and the significant Bartlett’s test of sphericity p < .001 (Sig. = 0.000) indicated that the sample was appropriate for confirmatory factor analysis in the future study.

The Cronbach Alpha Coefficient value of the scale indicated good to excellent levels of internal consistency in the subscales, and high reliability of the instrument as well [42].

To further test the relationship between the items used to create the three main constructs, a series of Pearson product-moment correlation coefficients were calculated. There was a significant positive relationship between all variables, with correlations ranging between .680 and .841, which indicates a strong relationship but does not indicate multicollinearity. This, therefore, provides further support for the reliability of the PLES-AA.

**Fig 3:**
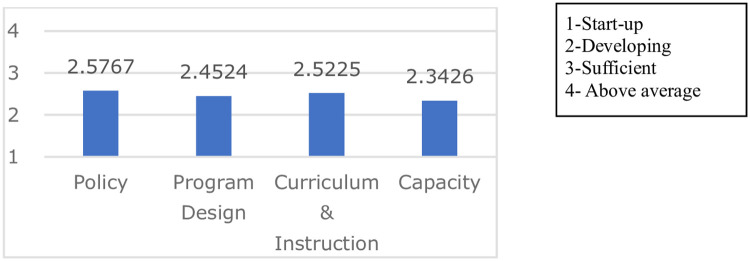
Current situation of PLE implementation in higher education.

The current situation of the PLE application in tertiary education in China was mainly in the stage of development (see Fig 3 below).

The above figure shows that the weighted average of the four categories was quite similar, ranging from 2.3 to 2.6, which indicated the developing stage of PLE implementation in higher education in China. As for the Policy category, 1.1 “Provide a rationale for the implementation of PLE in Higher Education” rated the highest (2.74), while 1.3 “The PLE platform provides a distribution policy for distributing organizational roles and responsibilities (teacher team building; responsibility distribution; role; resources)” scored the lowest (2.46). Concerning program category, 2.4 “The PLE platform learning network (forum, blog, discussion boards, etc.) supports students’ academic progress and social well-being” was comparatively well done in Chinese higher education (2.59), while 2.3 “The PLE platform makes consistent efforts to communicate the program mission, goals, and objectives to all stakeholders” (2.37) needed more effort. In terms of curriculum and instruction category, 3.1 “The PLE platform curriculum is aligned to Higher Education standards and performance goals” scored the highest (2.6), while 3.3 “The PLE platform can provide accreditation assessment and assurance of learning portfolios” was comparatively the lowest (2.46). Regarding the capacity category, 4.4 “The PLE platform supports and recommends personalized learning tools” was slightly ahead (2.4), while 4.2 “The PLE platform can support course-related resources in accumulation that are accessible for the length of years of the students’ education” needed more effort (2.29).

## 7. Discussion

Based on the existing online learning academic administrative scales and literature review, the five experts and the focus group of six administrative staff put forward extensive suggestions and comments for the revision of the PLES-AA. The CFA produced a four-factor, 16-item scale. The reliability test for the four dimensions revealed that the reliability coefficient alpha value for variable A was 0.869, for variable B it was 0.910, for variable C it was 0.875, and for variable D it was 0.859. The Cronbach’s alpha coefficient for each latent variable met the basic criteria of being greater than 0.7. It can be seen that the scale had good reliability.

The combined reliability CR and convergent validity AVE values were then calculated from the factor loading values of the validation factor analysis. The combined reliability CR was greater than 0.7, indicating that all the measures in each latent variable can consistently explain the latent variable.

The convergent validity of each dimension is reflected by the average variance extracted (AVE) value, which is usually used to reflect the convergent validity of the scale and can directly show how much of the variance explained by the latent variables are from the measurement error. The data showed that the AVE values were above the standard value of 0.5, which indicated that the scale had good convergent validity.

As for the current situation of PLE implementation in Chinese higher education, the survey results revealed that the majority of the participants were familiar with the concept of PLE, and PLE in Chinese tertiary education was at a developing stage. This indicated that: first, growing attention had been paid to PLE, which was no longer alien to teachers and staff in higher education in China; second, the standards of PLES-AA in higher education were being developed.

The development stage was consistent with the status quo of PLE research in China, which was still in its infancy. First, the PLE concept still needs consensus understanding; second, the collaboration among all the stakeholders involved in the implementation of PLE, including teachers, learners, governments, etc., who may take a long time to adjust to the new roles, [43, 44] new teaching and learning philosophy [45–47] and literacy [48] and a reform of educational policies as a whole [49, 50]. Third, the crisis and risks of the new technologies bring to the education field, including data privacy and protection, data storage, the proper use of learning portfolios, etc [51–53].

The realization of personalized learning for digital natives was just around the corner, thanks to the rapid development of information, communication, and technology (ICT) and the emergence of new educational theories. Researchers, parents, government officers, and ICT experts need to collaborate and cooperate in policy, economy, society, and technology (PEST) to create a collaborative environment for the implementation of PLE in higher education.

## 8. Conclusion and limitation

In the digital era, universities are changing rapidly to meet the demands of a new clientele that are more flexible, personalized, and diversified. However, even though e-learning systems are widely applied, there is still a lack of research supporting a model to guide personalized educational reforms. Limited efforts have been made to develop an assessment tool for measuring the readiness of academic administrations in higher education to implement PLEs. The current investigation is one of the few, if not the first, studies that construct and validate the PLE scale from an academic administrative perspective (PLES-AA) to establish quality assurance for PLE implementation in higher education.

This study contributes to current research in PLEs by advancing the theoretical understanding of PLEs’ implementation in higher education from the academic administrative perspective. Additionally, a pilot measurement scale (PLES-AA) gives fresh insight into measuring the readiness of the academic administration in higher education. The current situation survey indicated a developing stage of PLE in tertiary education in China.

Concerning the study’s limitations, the first is that the small size of our sample, compared to a larger sample, would be associated with lower statistical power for statistical significance testing, resulting in less accurate estimates of the underlying population. Second, to further validate the PLES-AA, more academic administrative staff could be involved in future research.

Based on the methodology used and the results obtained, it can be indicated that the validated PLE scale from an academic administrative perspective meets the requirements necessary to ensure the validity of its content, internal consistency, and reliability. It can also be generalized to other online or virtual learning situations as long as they have characteristics similar to the PLE. As for future research, the PLE scale from technological, teaching, and learning perspectives could be developed to guarantee the successful implementation of PLE in higher education.

## Supporting information

S1 Appendix (DOCX)

S1 Data (XLSX)
