# Supplementary material for: Development and validation of a PLE scale from academic administrative perspective (PLES-AA) in tertiary education: A pilot study in China
Source: PLoS One. 2022 Aug 5;17(8):e0272214. doi: 10.1371/journal.pone.0272214 (PMC9355211; doi:10.1371/journal.pone.0272214)
Supplement: S1 Appendix — (DOCX) [file pone.0272214.s001.docx]

Appendix 1

The PLE scale from Academic Administrative Perspective (PLES-AA)

| **Policy: The criteria in this section intends to ensure there is both a rational and relevant system for the implementation of a PLE platform consistent with overall HE standards and policies.** | | | |
| --- | --- | --- | --- |
| 1.1 Provide rationale for the implementation of PLE in HE. | 1.2 The PLE platform provides implementation planning policies to address policy support and flexibilities, funding sources and technology, and infrastructure (budget plan, technology support..). | 1.3: The PLE platform provides a distribution policy for distributing organizational roles and responsibilities (teacher team building; responsibility distribution; role; resources). | 1.4 The PLE platform provides Quality Assurance for monitoring and evaluating (teacher training and academic research; teaching and learning monitoring and evaluation; learning portfolio, project monitoring). |
| **Program Design: The criteria in this section reflect the PLE platform’s mission and goals and addresses the accessibility and understanding of these to relevant stakeholders, including students, parents, and community members.** | | | |
| 2.1 The PLE platform mission statement reflects the importance of the PLE and is informed by HE research bases and practices. | 2.2 The PLE platform goals and objectives are clearly defined, specific, measurable, achievable, results-focused, and time-bound. | 2.3 The PLE platform makes consistent efforts to communicate the program’s mission, goals, and objectives to all stakeholders. | 2.4 The PLE platform learning network (forum, blog, discussion boards…) supports students’ academic progress and social well-being |
| **Curriculum/Instruction: The criteria in this section reflect the design and rigour of the PLE platform curriculum policies and the reliability of its implementation.** | | | |
| 3.1 The PLE platform curriculum is aligned to HE standards and performance goals. | 3.2 The PLE platform curriculum is monitored for quality and reliability. | 3.3 The PLE platform can provide accreditation assessment and assurance of learning portfolios. | 3.4 The PLE platform system can comply and meet the criteria for learning technology interoperability for external and internal learning services. |
| **Capacity: The criteria in this section reflect the ability and capacity of a PLE platform that can support systematic capacities and infrastructure concurrently. The specific operation or implementation method is not included in the scope of this description.** | | | |
| 4.1 The PLE platform can provide courses in accumulation that are accessible for the length of years of the students' education. | 4.2 The PLE platform can support course-related resources in accumulation that are accessible for the length of years of the students’ education. | 4.3 The PLE platform can support previous platform users as well as current platform users | 4.4: The PLE platform supports and recommends personalized learning tools. |
